# A systematic review and *meta*-analysis of the efficacy of N95 respirators and surgical masks for protection against COVID-19

**DOI:** 10.1016/j.pmedr.2023.102414

**Published:** 2023-09-12

**Authors:** Gaohong Wu, Qingyang Ji, Yuan Shi

**Affiliations:** a Department of Neonatology, Zhuhai Center for Maternal and Child Health Care, Zhuhai, China; b Department of Breast Surgery, Zhuhai Center for Maternal and Child Health Care, Zhuhai, China; c Department of Neonatology Children’s Hospital of Chongqing Medical University, National Clinical Research Center for Child Health and Disorders, Ministry of Education Key Laboratory of Child Development and Disorders, Chongqing Key Laboratory of Pediatrics, Chongqing, China Chongqing, China

**Keywords:** COVID-19, SARS-CoV-2, N95 respirators, Surgical masks, Meta-analysis

## Abstract

Former *meta*-analyses concluded that there was not sufficient evidence to determine the effect of surgical masks and N95 respirators. We collected randomized controlled trials (RCTs) and conducted a systematic review and *meta*-analysis to evaluate the efficacy of N95 respirators and surgical masks for protection against COVID-19. We retrieved relevant RCTs published between January 2019 and January 2023 by searching the PubMed, EMBASE, and Cochrane CENTRAL. Study quality was evaluated using the Cochrane Risk of Bias tool with the RevMan 5.4 software. Meta-analyses were conducted to calculate pooled estimates using the RevMan 5.4 software. A total of six RCTs were finally included. The findings revealed that wearing a mark made little difference in preventing COVID-19 [odds ratio (OR) = 0.10; 95% confidence interval (CI): 0.01–0.93; *P* = 0.04]. Subgroup analysis showed that the heterogeneity of data was I^2^ = 64% (OR = 0.32; 95% CI: 0.06–1.77; *P* = 0.19) for surgical mask use and I^2^ = 0% (OR = 0.03; 95 %CI: 0.01–0.15; *P* < 0.01) for N95 respirator use. The heterogeneity of data for medical staff was I^2^ = 0% (OR = 0.03; 95 %CI: 0.01–0.12; *P* < 0.01). Meta-analysis indicated a protective effect of N95 respirators against COVID-19, particularly for medical staff. The use of surgical masks is not associated with a lower risk of COVID-19. However, the subgroup using N95 respirators, particularly medical staff, showed a significant protective. These findings suggest that N95 respirators should be reserved for high-risk medical staff in the absence of sufficient resources during an epidemic. But the number of included studies was small, more studies in future analyses is required to reduce the risk of distribution bias.

## 1 Introduction

Respiratory infectious diseases are characterized by high infectivity and rapid epidemic contagion via multiple difficult-to-control transmission channels (Shi et al., 2017). The severe acute respiratory syndrome coronavirus 2 (SARS-CoV-2) outbreak, which originated from Wuhan, China, has become a major global health issue. This novel coronavirus can cause severe respiratory tract infections and lead to bronchiolitis or pneumonia. The disease and presentations resulting from this infection have been designated as coronavirus disease 2019 (COVID-19) by the World Health Organization (WHO) on February 12, 2020. The high prevalence of SARS-Cov-2 infections led the WHO to declare this an international public health emergency on January 30, 2020 (Huang et al., 2020). At present, there are no specific drugs for COVID-19; however, many public health measures have been implemented to improve disease control and prevention. Among these measures, face masks have been easily available and accessible since the early stage of the outbreak.

Surgical masks and N95 respirators are personal protective equipment used by medical staff and have been considered highly significant for the prevention of SARS-Cov-2 (Lancet, 2020). Nevertheless, there is not sufficient evidence establishing that the use of surgical masks and N95 respirators reduces SARS-Cov-2 spread. Furthermore, N95 respirators may play a limited role in low-resource circumstances wherein there could be limited supply or unaffordability of N95 respirators (Chughtai et al., 2013). The WHO recommends using N95 respirators in high-risk situations and surgical masks in low-risk situations, but the Centers for Disease Control and Prevention recommends using N95 respirators in both high- and low-risk situations (World Health Organization (WHO), 2007, Center for Disease Control and Prevention (CDC), 2023). Owing to a small number of studies and the consequent deficit in statistical power, previous *meta*-analyses have also not identified sufficient evidence to determine the effect of N95 respirators and surgical masks (Smith et al., 2016, Offeddu et al., 2017). Notably, more rigorous RCTs comparing the effectiveness of N95 respirators and surgical masks in preventing COVID-19 have been published in recent years, and these were not included in former *meta*-analyses (Bundgaard et al., 2021, Varela et al., 2022).

In this study, we conducted a comprehensive *meta*-analysis of the effectiveness of N95 respirators and surgical masks for protection against COVID-19. We believe that the findings will provide a scientific basis for the formulation of policies related to the use of surgical masks and N95 respirators.

## 2 Methods

### 2.1 Systematic review registration

This systematic review was registered with number CRD42020179966 (https://www.crd.york.ac.uk/PROSPERO).

### 2.2 Disclosure of ethical compliance

All analyses in this systematic review and meta- analysis were based on previous published studies that met ethical guidelines.

### 2.3 Search strategy

Articles published in English between January 2019 and January 2023 that explored the relationship between wearing surgical masks or N95 respirators and protection against COVID-19 were retrieved from Cochrane CENTRAL, PubMed, EMBASE, and Web of Science databases. The following search terms were used: “SARS-Cov-2,” “COVID-19,” “RCTs,” “prevention,” “Surgical masks,” and “N95 respirators.” Logical operators (NOT, OR, and AND) were used to combine subject words and keywords (Table 1).

**Table 1:** Search strings for the four databases.

**Database**	**Search string**
PubMed	(masks [MeSH Terms] OR mask [title/abstract] OR N95 respirators [title/abstract] OR “surgical masks” [title/abstract] OR “face masks” [title/abstract] OR “surgical masks” [title/abstract] OR “surgical masks” [title/abstract] OR “surgical masks” [title/abstract] OR “surgical masks” [title/abstract] OR “surgical facemask” [title/abstract] OR “surgical facemasks” [title/abstract] OR “surgical face mask” [title/abstract] OR “surgical face masks” [title/abstract] OR SARS-Cov-2 [title/abstract] OR COVID-19 [title/abstract] OR prevention [title/abstract] OR control [title/abstract] OR measure [title/abstract] OR evaluate [title/abstract] OR effect [title/abstract] OR Public Health [title/abstract] OR medical staff [title/abstract] OR non-medical staff [title/abstract]
EMBASE	(“SARS-Cov-2”: abstract, title OR “COVID-19”: abstract, title) AND (“Public Health”: abstract, title OR “medical workers”: abstract, title OR “nursing home patient”: abstract, title) AND (“prevention”: abstract, title OR “control”: abstract, title OR “measure”: abstract, title OR “evaluate”: abstract, title OR “effect”: abstract, title OR “prevent”: abstract, title OR “control”: abstract, title OR “intervention”: abstract, title OR “outcome”: abstract, title)
Web of Science	TS=(mask OR facemask OR “face mask” OR “face masks” OR “medical” OR “surgical mask” OR surgical “masks” OR “medical facemask” OR “medical facemasks” OR “medical face mask” OR “medical face masks” OR “N95” OR “N95 respirators” OR “surgical facemask” OR “surgical facemasks” OR “surgical face mask” OR “surgical face masks” OR Infectious Diseases OR SARS-Cov-2 OR “COVID-19” OR “prevention” OR “control” OR “prevention and control” OR PPE OR “measure” OR “evaluate” OR “effect” OR “Public Health” OR “medical workers”) AND TS=(“healthcare worker” OR “healthcare workers” OR “health care worker” OR “health care workers” OR “health-care worker” OR “health-care workers” OR “healthcare professional” OR “healthcare professionals” OR “health care professional” OR “health care professionals” OR “health-care professional” OR “health-care professionals” OR staff OR “healthcare personnel” OR “health care personnel” OR “health-care personnel”)
Cochrane CENTRAL	SARS-Cov-2 OR COVID-19 in title, abstract, and keywords AND “surgical masks” OR “N95 respirators” OR “mask” in title, abstract, and keywords AND practice OR control OR measure OR evaluate OR effect OR prevent OR prevention and control OR intervention OR outcome in title, abstract, and keywords. Publication year was 2019–2023 for the trials.

Note: COVID-19: Coronavirus disease 2019; SARS-Cov-2: Severe acute respiratory syndrome coronavirus 2.

### 2.4 Inclusion criteria

Articles that met the following criteria were selected: (1) the study was a peer-reviewed RCT; (ii) the population was medical staff and/or non-medical staff; (iii) the exposure of interest was wearing surgical masks or N95 respirators; (iv) the outcome of interest was the proportion of infected patients in the experimental and control groups; and (v) the individuals had a diagnosis of COVID-19 supported by epidemiological history, clinical manifestations, and laboratory examinations (Yüce et al., 2021).

### 2.5 Exclusion criteria

We excluded guidelines, editorials, public press articles, reviews, publications with raw data unavailable, theoretical models, and articles published in languages other than English.

### 2.6 Data extraction

Data extraction was carried out in two steps. First, the literature was screened by two researchers according to the inclusion criteria. Second, the screened literature was then searched and evaluated by two other researchers according to the exclusion and inclusion criteria. To prevent inconsistencies, a pre-designed form was used to select study characteristics, baseline patient characteristics, and outcomes and definitions included in the literature, and any inconsistencies in recommendations were resolved by consultation with a third researcher. The main data extracted were as follows: the number of medical staff or non-medical staff who wore a mask and those who did not wear a mask.

### 2.7 Primary outcome

Numbers of COVID-19 cases in the comparisons (face masks *vs*. no face masks and N95 respirators *vs*. surgical masks).

### 2.8 Secondary outcome

Numbers of COVID-19 cases in the comparison between medical staff and non-medical staff.

### 2.9 Literature quality assessment

We used the Cochrane Risk of Bias tool (Produced by Cochrane Collaborative Network) to evaluate the quality of the methodology used in the included studies (Michaelis et al., 2018). The quality of RCTs was evaluated using the RevMan 5.4 software. The risk of bias was evaluated from six perspectives: measurement bias, attrition bias, reporting bias, choice bias, performance bias, and other biases (Table 2). According to the criteria for high risk, unclear risk, and low risk, the quality of the included studies’ methodology was divided into three levels as follows: mild bias: four or more of the above six items are low risk; moderate bias: two or three of the above six items are low risk; and severe bias: none or only one of the above six items is low risk.

**Table 2:** Cochrane risk of bias assessment form.

**Evaluation items**	**Evaluation content**	
Choice bias	Random sequence generation	The method of generating random assignment sequence is described in detail, which is convenient to evaluate the comparability between the groups.
	Assignment hidden	The method of hiding random distribution sequence is described in detail, which is convenient for judging whether the distribution of intervention measures can be predicted.
Performance bias	Blind method for researchers and subjects	The method of blinding used to prevent researchers and subjects from knowing the intervention measures is described in detail. This provides information that can be used to judge whether the blinding method is effective.
Measurement bias	Blind evaluation of research results	The method of blinding used to prevent the evaluators of the research results from knowing the intervention measures is described in detail. This provides information that can be used to judge whether the blinding method is effective.
Attrition bias	Integrity of result data	Data for each major outcome indicator, including those of subjects who were lost to follow-up or withdrew from the study, are reported completely. Data pertaining to subjects who were lost to follow-up or withdrew, the total number of people in each group (compared with the total number of randomly enrolled people), and the reasons for the loss of interview/withdrawal are clearly reported, which facilitates the assessment of the relevant treatment by the system evaluator.
Reporting bias	Selective reporting of research results	The information described can be used by system evaluators to judge the possibility of selective reporting of research results and relevant information.
Other biases	Other sources of bias	In addition to the above biases, the information provided can be used to assess the existence of other bias factors. If a question or factor is mentioned in the plan, corresponding answers are required.

### 2.10 Statistical methods

RevMan 5.4 software provided by the Cochrane Collaboration was used to conduct this *meta*-analysis of the proportion of individuals using medical masks between the experimental and control groups. I^2^ tests and Q were used to evaluate the included studies; heterogeneity (the Q test is the traditional method used in *meta*-analysis of heterogeneity; I^2^ tests can measure the degree of difference among multiple research effects and can describe the percentage of variation caused by inter research in the total variation). When *P* > 0.1 and *I*^2^ ≤ 50%, a fixed-effects model was used to merge data; when *P* < 0.1 or *I*^2^ > 50%, a random-effects model was used to merge data. We used the odds ratio (OR) and 95% confidence interval (CI) to express enumeration data. *P* < 0.05 indicates a statistically significant difference.

**Fig. 1:**
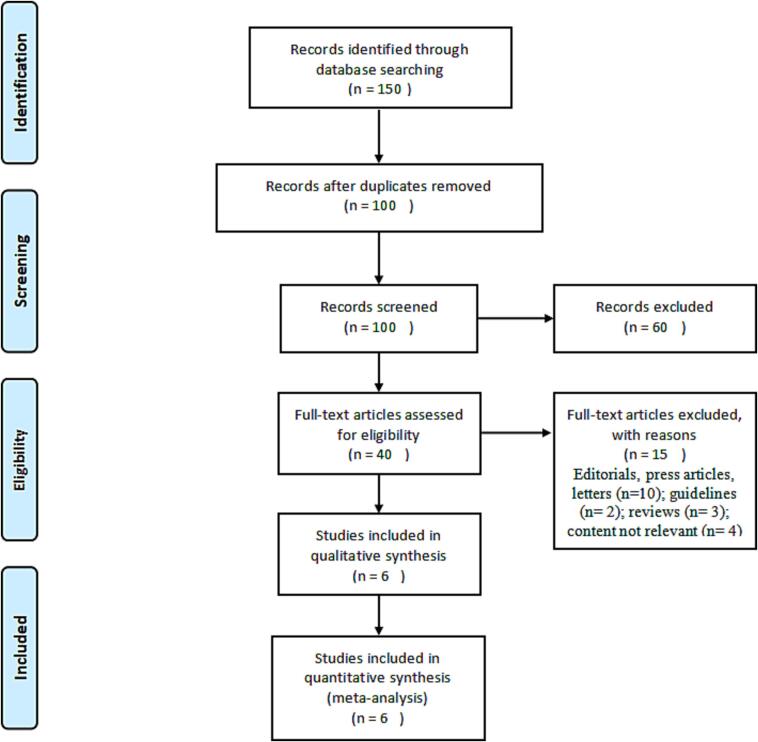
Summary of the literature search and inclusion process.

Document retrieval flow chart **(**Fig. 1**)**.

## 3 Results

### 3.1 Literature search results

**Fig. 2:**
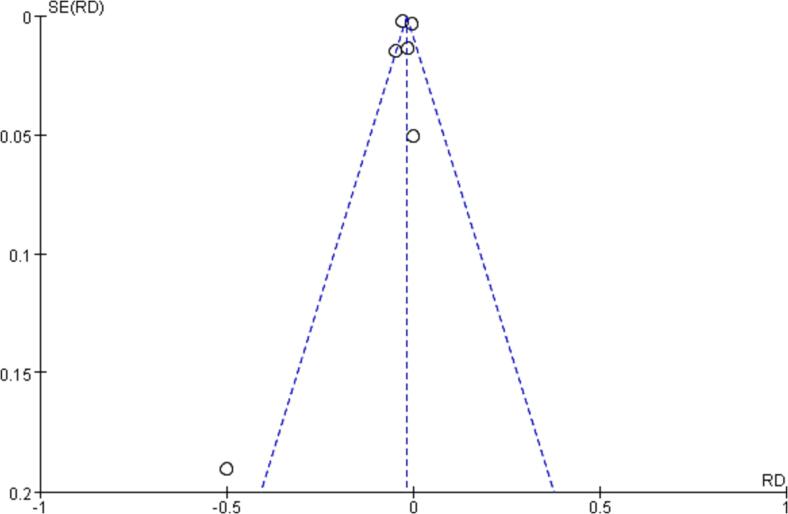
RCTs reporting the effect of face mask versus no face mask. **Funnel plot assessing publication bias in RCTs investigating the effectiveness of face mask *vs*. no face mask for protection against COVID-19; Harbord’s estimated bias coefficient: −0.58; *P* = 0.599.**

After searching 150 articles from four databases, six articles were included after the final screening (Fig. 1). We searched the full text of 150 articles and excluded 144 that did not meet our inclusion criteria. Finally, we selected six RCTs (Table 3), among which three RCTs analyzed the effectiveness of N95 respirators and three RCTs analyzed the effectiveness of surgical masks for protection against COVID-19. There was no actual evidence indicating publication bias (Fig. 2). The assessment of the risk of bias of six RCTs using the RevMan 5.4 software showed mild overall bias (Fig. 3A, Fig. 3B).

**Table 3:** Summary of RCTs assessing masks' effectiveness for protection against COVID-19 (n = 6).

**Author**	**Setting**	**Year of publication**	**Study design**	**Virus types**	**Mask type**	**Face mask (n)**		**No face mask (n)**		**OR**	**95% CI**
						**Infected**	**Non-infected**	**Infected**	**Non-infected**		
Burke (xxxx). USA	Medical staff	2020	RCTs	SARS-Cov-2	N95	0	64	0	13	NE	NE
Heinzerling (Heinzerling et al., 2020). USA	Medical staff	2020	RCTs	ARS-Cov-2	Surgical	0	31	3	3	0.02	0.00–0.37
Wang (Wang et al., 2020). China	Medical staff	2020	RCTs	ARS-Cov-2	N95	0	278	10	205	0.04	0.00–0.60
Wang (Wang et al., 2020). China	Medical staff	2020	RCTs	ARS-Cov-2	N95	1	1285	119	3917	0.03	0.00–0.18
Bundgaard (Bundgaard et al., 2021). Denmark	Non medical staff	2021	RCTs	ARS-Cov-2	Surgical	42	2350	53	2417	0.82	0.54–1.23
Varela (Varela et al., 2022). Colombia	Non medical staff	2022	RCTs	ARS-Cov-2	Surgical	1	140	3	139	0.33	0.03–3.22

Note: NE: Not estimable; SARS-Cov-2: Severe acute respiratory syndrome coronavirus 2; RCTs: Randomized controlled trials; OR: odds ratio; CI: confidence interval.

**Fig. 3A:**
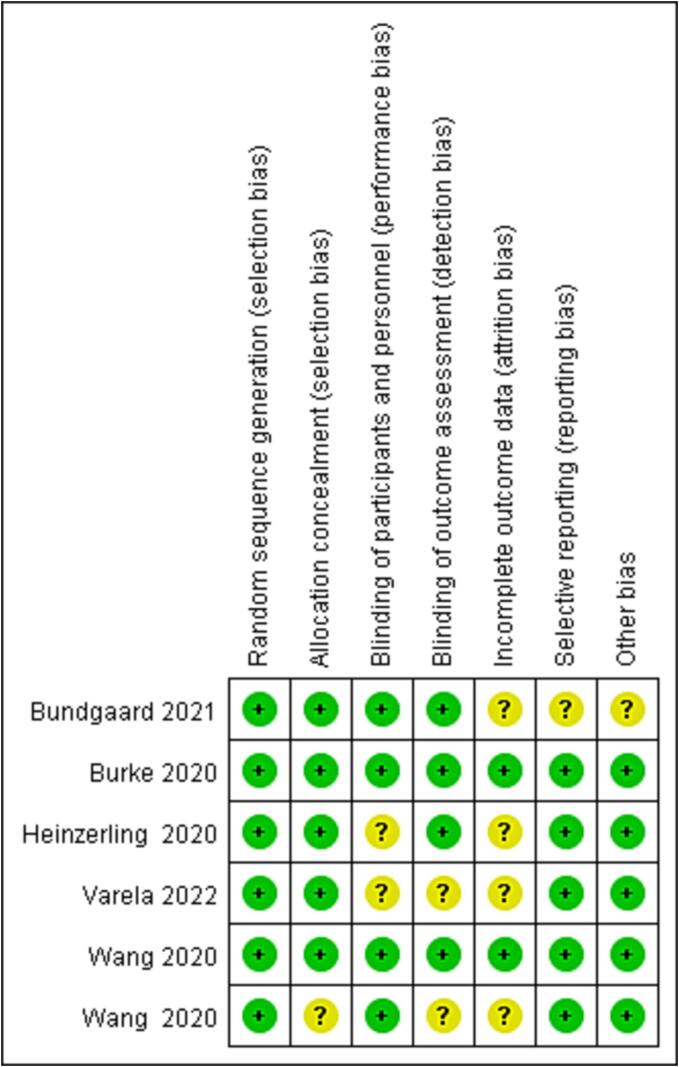
RCTs received a high (red), low (green), or unclear (yellow) risk of bias score for each domain.

**Fig. 3B:**
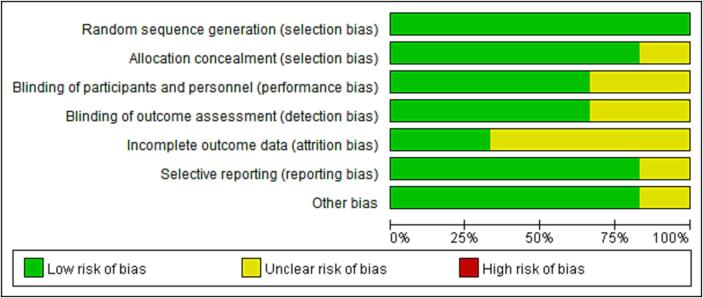
Percentage of RCTs with high, low, or unclear risk of bias in each domain.

### 3.2 Face mask use *vs*. No face mask use for protection against COVID-19

#### 3.2.1 Primary outcome

Pooling of all six RCTs revealed an estimate of the effect of wearing a face mask on the outcomes of COVID-19 cases (OR = 0.10; 95% CI: 0.01–0.93; *P* = 0.04; low-certainty evidence), suggesting that wearing a mask may make little difference for this outcome (Fig. 4A). Because of the heterogeneity, data were divided for subgroup analysis according to surgical mask use and N95 respirator use. Subgroup analysis showed that the heterogeneity of data was I^2^ = 64% (OR = 0.32; 95% CI: 0.06–1.77; *P* = 0.19; low-certainty evidence) for surgical mask use and I^2^ = 0% (OR = 0.03; 95% CI: 0.01–0.15; *P* < 0.01; high-certainty evidence) for N95 respirator use (Fig. 4B). These results showed that the heterogeneity of the data was very high for surgical mask use and very low for N95 respirator use. Therefore, the heterogeneity of data in the included studies was related to the differences in outcomes associated with the use of surgical masks and N95 respirators. Furthermore, wearing N95 respirators conferred significantly greater protection against COVID-19.

**Fig. 4A:**
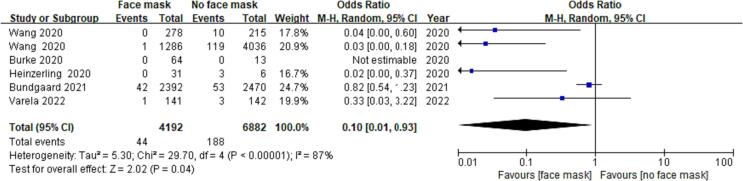
A *meta*-analysis of RCTs assessing the protective effects of N95 respirators and surgical masks against COVID-19.

**Fig. 4B:**
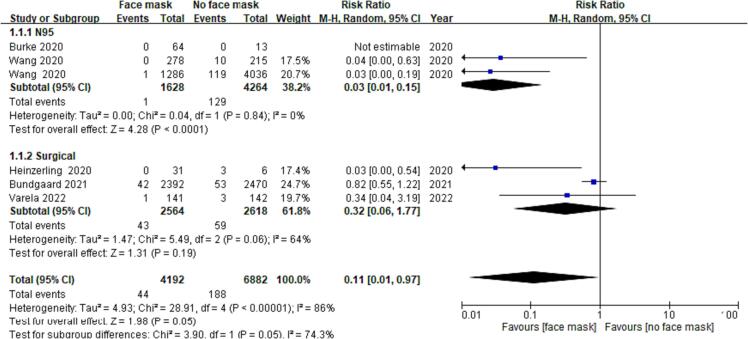
Subgroup analysis of the outcomes of wearing N95 respirators and surgical masks for protection against COVID-19.

#### 3.2.2 Secondary outcome

That heterogeneity of data according to subgroup analysis was I^2^ = 0% (OR = 0.03; 95% CI: 0.01–0.12; *P* < 0.01; high-certainty evidence) for medical staff and I^2^ = 0% (OR = 0.80; 95% CI: 0.54–1.18; *P* = 0.26; low-certainty evidence) for non-medical staff (Fig. 4C). These results showed that the difference in heterogeneity of data was insignificant between medical staff and non-medical staff. Therefore, the heterogeneity of data in the included studies had little association with the differences in outcomes of medical and non-medical staff. Furthermore, medical staff have a stronger awareness of the ways to be protected from COVID-19.

**Fig. 4C:**
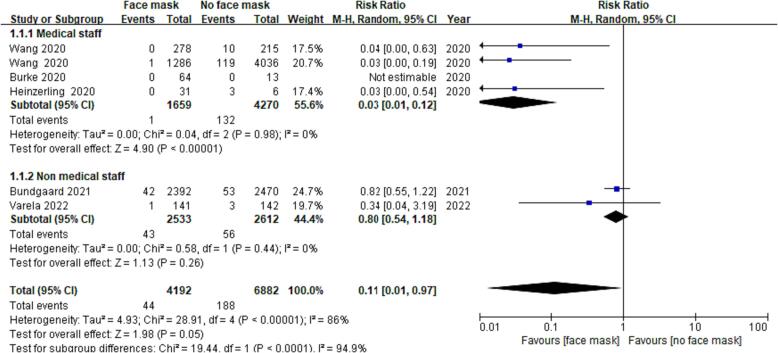
Subgroup analysis of the outcomes of medical staff and non-medical staff for protection against COVID-19.

## 4 Discussion

The pooled estimates of effects from RCTs for wearing and not wearing masks suggest little difference in interrupting COVID-19′s spread; however, the confidence intervals were wide, and there was considerable heterogeneity. This heterogeneity may be attributable to differences in the inclusion and exclusion criteria among the studies. Our findings for N95 respirator and surgical mask subgroups revealed a significantly higher protective effect of using N95 respirators over surgical masks, and this is consistent with previous publications (Liang et al., 2020, Nanda et al., 2021). However, there was no difference between using surgical masks and using no face masks in terms of protection against COVID-19, and this is not consistent with previous publications (Liang et al., 2020). Between medical staff and non-medical staff subgroups, we found the incidence of COVID-19 to be relatively low in medical staff; this could be due to the stricter protective measures taken by medical personnel (Manoj et al., 2021).

Given the ongoing COVID-19 pandemic and recurrent outbreaks of novel strains, numerous face masks may be required to be worn for long periods to prevent infections (Jefferson et al., 2020). National and international guidelines unanimously recommend the use of N95 respirators for protection against aerosols; however, this is inconsistent with the current recommendations for non-aerosol prophylaxis and routine care for COVID-19 patients (Wang and Ratner, 2020, Dheda et al., 2021, Mahmood et al., 2020, Science et al., 2021). Surgical masks are cheaper than N95 respirators; however, the European Centers for Disease Control and Prevention and the Centers for Disease Control and Prevention recommend N95 respirators over surgical masks for non-aerosol-generating procedures, particularly for medical staff (European Centre for Disease Prevention and Control, 2020). Indeed, Kobayashi et al. reported that long-term use and reuse of N95 respirators during the COVID-19 pandemic could effectively protect volunteer workers (Kobayashi et al., 2020). Although N95 respirators may offer better protection than surgical masks against COVID-19, in real-world practice, routine use of N95 respirators seems less acceptable because of markedly more discomfort associated with wearing them, which is often associated with issues like headaches (Offeddu et al., 2017). However, notably, surgical masks are primarily designed to protect the environment from the wearer, whereas N95 respirators are aimed at protecting the wearer from the environment (Bałazy et al., 2006). Contrary to this distinction, the current WHO recommend the use of N95 respirators during the care of patients with COVID-19 (Infection prevention and control in the context of coronavirus disease, 2019). The observed lack of surgical mask that wears in interrupting SARS-CoV-2′s spread in our *meta*-analysis has a lot of potential reasons, such as poor study design, lower compliance with mask wearing (particularly among children), the quality of the masks used, self-contamination of the mask by hands, lack of protection in terms of eye exposure to respiratory droplets, and risk compensation behavior leading to an exaggerated sense of security. Our *meta*-analysis showed that the efficacy of COVID-19 prevention did not statistically significantly differ with using surgical masks and using no face masks. Therefore, the efficacy of surgical masks for protection against COVID-19 remains to be fully clarified.

This *meta*-analysis has some limitations. First, the number of included studies was small and may thus involve distribution bias. Inclusion of a greater number of studies in future analyses is required to reduce the risk of distribution bias. Second, there may be publication bias in the included articles, and we cannot assess it because the RCTs included were too few. Third, heterogeneity was identified among the data of the included studies, which may be attributed to the type of the mask. In this study, although subgroup analysis of the use of surgical masks and N95 respirators was conducted for some indicators, it was not conducted for different regions and other types of masks. More detailed subgroup analyses are therefore required to reach more convincing conclusions. Finally, not in all trials was the infection source identified, and some medical staff may have been infected before the trial. Notably, in this study, we did not search for RCTs comparing the use of surgical masks with N95 respirators for protection against SARS-CoV-2 infection, and this issue is worthy of consideration.

## 5 Conclusions

Herein, a literature review and *meta*-analysis of RCTs on the protective effects of surgical masks and N95 respirators against COVID-19 was conducted. The RCTs included in this *meta*-analysis showed that the use of face masks made little difference in interrupting the spread of COVID-19. However, the subgroup showed a significant protective effect of using N95 respirators, particularly for medical staff. The findings suggest that in the absence of adequate resources during an epidemic, N95 respirators should be reserved for medical staff at high risk of infection.

## Declaration of Competing Interest

The authors declare that they have no known competing financial interests or personal relationships that could have appeared to influence the work reported in this paper.

## Data Availability

Data will be made available on request.
